# Optogenetic sensors and effectors: CHROMus—the Cornell Heart Lung Blood Institute Resource for Optogenetic Mouse Signaling

**DOI:** 10.3389/fphys.2014.00428

**Published:** 2014-11-06

**Authors:** Bo Shui, Jane C. Lee, Shaun Reining, Frank K. Lee, Michael I. Kotlikoff

**Affiliations:** Department of Biomedical Sciences, College of Veteirnary Medicine, Cornell UniversityIthaca, NY, USA

**Keywords:** Ca^2+^ sensors, fluorescent imaging, genetically encoded Ca^2+^ indicators, rhodopsin, green fluorescent proteins, transgenic mice

## Abstract

Significant progress has been made in the last decade in the development of optogenetic effectors and sensors that can be deployed to understand complex biological signaling in mammals at a molecular level, without disrupting the distributed, lineage specific signaling circuits that comprise nuanced physiological responses. A major barrier to the widespread exploitation of these imaging tools, however, is the lack of readily available genetic reagents that can be easily combined to probe complex biological processes. Ideally, one could envision purpose–produced mouse lines expressing optically compatible sensors and effectors, sensor pairs in distinct lineages, or sensor pairs in discrete subcellular compartments, such that they could be crossed to enable *in vivo* imaging studies of unprecedented scientific power. Such lines could also be combined with mice to determine the alteration in signaling accompanying targeted gene deletion or addition. In order to address this lack, the National Heart Lung and Blood Institute has recently funded an optogenetic resource designed to create optically compatible, combinatorial mouse lines that will advance NHLBI research. Here we review recent advances in optogenetic sensor and effectors and describe the rationale and goals for the establishment of the Cornell/National Heart Lung Blood Resource for Optogenetic Mouse Signaling (CHROMus).

## Introduction

Normal and abnormal physiological processes involve dynamic and highly specific cell signals that occur on a millisecond time scale. Most of these events are opaque to scientific observation in real time, however, because molecular scale information about these events is typically restricted to average measurements at a single point in time, or the study of reductive, *ex vivo* preparations of isolated cells that inadequately represent the more complex physiological processes of interest. The advent of the use of re-engineered fluorescent protein sensors and light–activated channel protein effectors under cell-specific transcriptional control in genetically modified organisms has enabled a new era of *in vivo* biology, in which increasingly powerful molecular detectors and actuators of cellular processes are used to understand complex biological events in real time, *in vivo*, in mammals.

**Figure 1:**
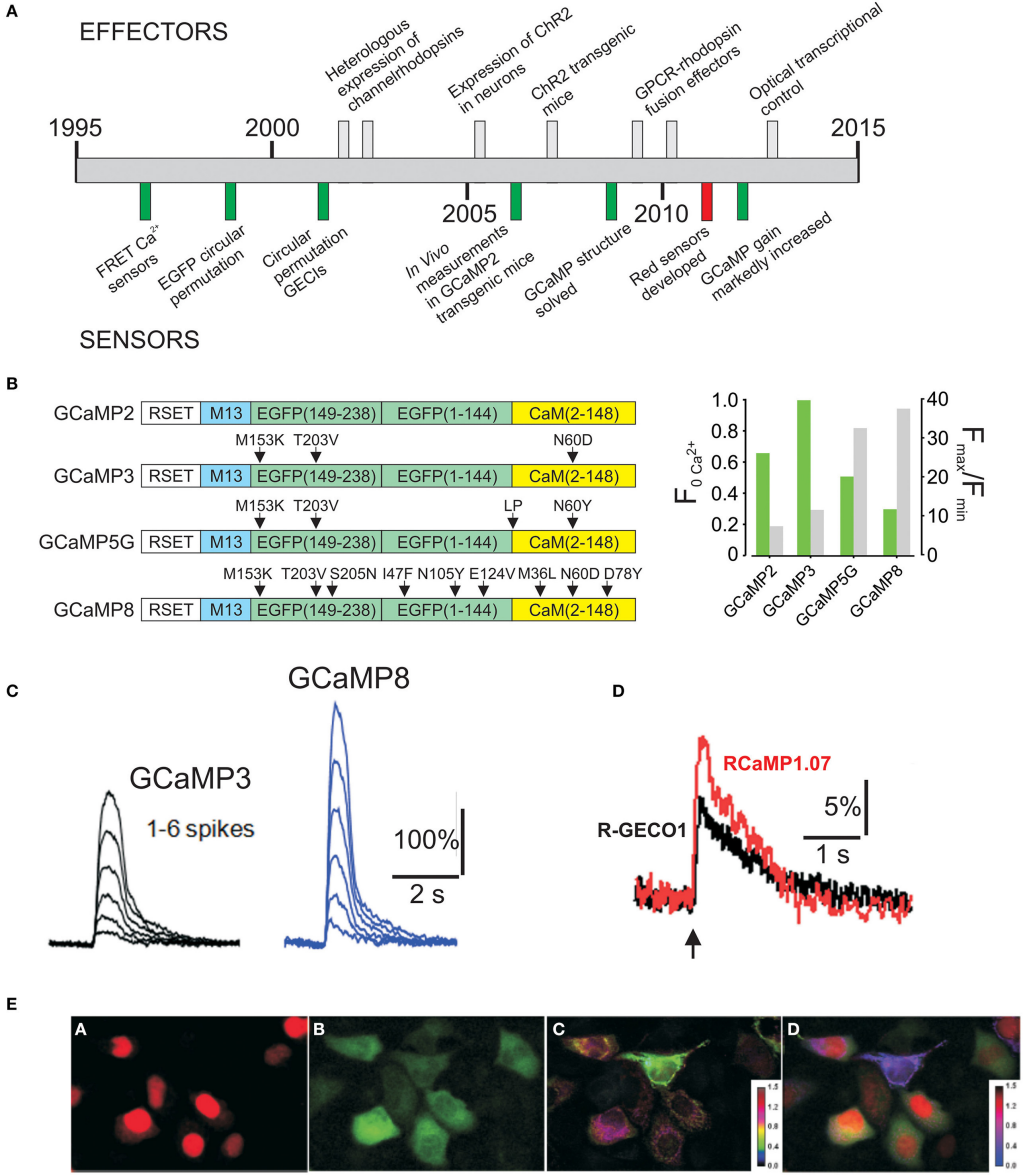
**(A)** Timeline of development of optogenetic effectors and sensors. **(B)** Evolution of GCaMP2 improving basal fluorescence (F_0Ca^2+^_) and dynamic range. **(C)** Improved performance of GCaMP8 to spike depolarizations in cultured neurons. Images from Ohkura et al. (2012a) **(D)** Response of R-GECO1 and RCaMP1.07 to a single spike. Images from Ohkura et al. (2012b) **(E)** Simultaneous imaging with Red and Green GECIs. Images from Ohkura et al. (2012b).

The confluence of technological advances in purpose-engineered sensor and effector proteins, mouse genetics, and fluorescence imaging has introduced unprecedented opportunities for the study of complex biological processes *in vivo*, in real time, in mammals (Tsien, 2003; Muller-Taubenberger, 2006; Kotlikoff, 2007; Fenno et al., 2011; Mehta and Zhang, 2011; Miyawaki, 2011; Tangney and Francis, 2012). Optogenetic tools, including high gain molecular sensors, and efficiently coupled light–activated effectors can be used to interrogate complex biological processes in ways that are not possible with conventional methods, and parallel advances in bacterial artificial chromosome (BAC) transgenesis have enabled the production of mice that express high levels of optical sensors and effectors in a lineage specific manner without disrupting endogenous genetic loci (Lee et al., 2001). More than a decade of progress on genetically encoded Ca^2+^ indicators (Palmer and Tsien, 2006; Kotlikoff, 2007; Zhao et al., 2011; Chen et al., 2013) and light activated optogenetic effector proteins derived from bacterial or mammalian opsins (Deisseroth, 2011; Rein and Deussing, 2012) has resulted in the creation of a mature set of tools (Figure 1A). Thus, it is now possible to genetically engineer and cross mouse strains in which complex systems can be interrogated at a molecular level in mammals, in real time, *in vivo*, a requisite capability to understand the way that the interaction of millions of cells are coordinated to produce physiological outputs, or fail to do so.

The major barrier to the effective use of these technologies in cardiovascular biology, however, is the lack of mouse lines designed to probe clinically relevant, complex physiological, and pathological interactions. In this brief review, we describe a new resource established by the National Heart, Lung, and Blood Institute (NIH R24 HL120847-01) to accelerate the adaptation of these technologies. CHROMus, the Cornell/NHBLI Resource for Optogenetic Mouse Signaling (chromus.vet.cornell.edu), is designed to produce combinatorial mouse lines with discrete fluorescent characteristics, creating a library of reagents with compatible optical properties that can be effectively exploited for effector/detector and lineage/lineage signaling experiments relevant to heart, lung, and blood biology.

## Genetically encoded molecular sensors

Genetically encoded fluorescent sensors that respond dynamically to changes in concentration of cellular molecules have advanced dramatically since the first sensors were developed from fluorescent proteins (Miyawaki et al., 1997, 1999; Nakai et al., 2001). While the range of detected molecules has steadily advanced (Newman et al., 2011), the most progress has been made on genetically encoded Ca^2+^ indicators, or GECIs, which have been progressively improved and expanded to additional wavelengths, lower Ca^2+^ affinities, and ratiometric molecules (Zhao et al., 2011; Despa et al., 2014). GECIs measure the key molecular signal underlying cell functions such as heart, vessel, and airway contraction, lung secretion, autonomic neurotransmission, and immunocyte function (Clapham, 1995; Berridge et al., 1999). Thus, these tools enable *in vivo* and *in situ* experiments that simply could not be performed using synthetic reagents such as optical dyes, because of the need for lineage specificity. Moreover, once germline transmission is achieved, the degree of expression is consistent and is no longer an experimental variable. This approach has been applied to studies of cardiovascular, lung, and airway inflammation, cardiac and smooth muscle electrical and chemical signaling, endothelial barrier function and control of vascular tone, organellar signaling, tissue repair, and the functional evaluation of stem cell therapies. Mouse lines expressing genetically encoded Ca^2+^ indicators (GECIs) in cardiac musle (Tallini et al., 2006a), and endothelium (Tallini et al., 2007) have been used to unravel signaling critical for cardiac development (Tallini et al., 2006a), vascular control (Tallini et al., 2007; Ledoux et al., 2008; Bagher et al., 2011; Sonkusare et al., 2012, 2014), and cardiac cell therapy (Roell et al., 2007; Shiba et al., 2012), but the limited number of these lines has limited adaption of the technology. The lack of these reagents relates to several factors: they are time consuming and expensive to produce, require technology that is often not present in typical labs, and are difficult to justify on a single grant. In this brief chapter, we describe a recently funded NHLBI resource designed to address this lack by the creation of approximately 50 lines of mice in which third-generation genetic Ca^2+^ sensors and optogenetic effectors are specified in lineages relevant to cardiac, vascular, lung, and blood diseases.

GCaMPs and RCaMPs: GCaMP sensors constitute a robust platform of 3rd generation sensors with high gain, and variable gain and Ca^2+^ affinity (Nakai et al., 2001; Tallini et al., 2006a; Tian et al., 2009; Muto et al., 2011; Zhao et al., 2011; Despa et al., 2014). Structural and biophysical studies of GCaMP2 indicate that the Ca^2+^ -dependent fluorescence occurs through a Ca^2+^/Calmodulin -dependent de-protonation of the fluorophore that has been destabilized and rendered vulnerable to hydration and protonation by the circular permutation of EGFP (Wang et al., 2008). In the protonated state (e.g. at low pH), the fluorophore has an absorbance peak at 395 nm, and minimal absorption and fluorescence at 488 nm excitation; circular permutation of the original EGFP results in an almost completely protonated fluorophore at physiological pH. However, Ca^2+^ binding to the fused calmodulin domain of GCaMPs eliminates proton (water) access to the fluorophore, resulting in a rapid deprotonation and an attendant spectroscopic shift to bright fluorescence at 488 nm excitation. Structural analysis indicates that the phenolic oxygen in GCaMP2- Ca^2+^ is stabilized in the anionic state through the formation of hydrogen bonds with Thr-116 and Ser-118(Wang et al., 2008). Thus, GCaMP molecules that are not Ca^2+^ bound have an absorbance maximum of 399nm and shift rapidly to 488 nm absorption upon Ca^2+^ binding.

GCaMPs have also been markedly improved since the development of GCaMP2 (Tallini et al., 2006a) (Figures 1B,C), which itself was an improvement in the stability and dynamic range of the original GCaMP1(Nakai et al., 2001), and enabled the first *in vivo* Ca^2+^ measurements in a transgenic mouse line (Tallini et al., 2006a). Three additional mutations in GCaMP2 (M153K, T203V in eGFP, and N60D in calmodulin) produced GCaMP3, with higher brightness at Ca^2+^ saturation and a somewhat higher dynamic range than GCaMP2 (Tian et al., 2009). Recently Campbell's laboratory identified several mutations through a large scale mutational screen in bacteria, leading to G-GECO1, which has an increased dynamic range, but some loss of brightness (Zhao et al., 2011). Over the past year two additional significant improvements have been reported. Akerboom et al. conducted an extensive structure-guided analysis, leading to improvements in the dynamic range of GCaMP3; several GCaMP5s were reported, including GCaMP5G, which improves the dynamic range of GCaMP3 almost 3 fold while decreasing minimum brightness only modestly (Akerboom et al., 2012). Experiments from Nakai's laboratory introduced EGFP superfolding mutations in GCaMP2, producing GCaMP-HS (Muto et al., 2011), and used site directed and random mutations to produce GCaMP6, GCaMP7, and GCaMP8 (Ohkura et al., 2012a). GCaMP5G and GCaMP8 are the highest dynamic range GCaMPs developed to date (F/F0 = 32.7 and 37.5, respectively), with GCaMP8 having a modestly higher dynamic range and more rapid off-kinetics, but less brightness at basal Ca^2+^, an issue that is of less concern in stable transgenic lines with consistent expression.

Red–shifted indicators provide an important advantage to GCaMPs in the context of determining cell-cell signaling, and when partnered with rhodopsins whose excitation spectra overlap with GCaMPs. Important advances in red–shifted indicators have also been made. Zhao et al. circularly permutated mApple and conducted a bacterial screen that finally resulted in R-GECO1 (Zhao et al., 2011). This red–shifted indicator has a distinct spectrum relative to GCaMPs, a 16 fold dynamic range, and brightness that exceeds GCaMP2. R-GECO1 has been improved to R-CaMP1.07 by Nakai's group with an F/F0 of 28.7 and slightly higher brightness (Ohkura et al., 2012b) (Figure 1D). Importantly, the spectrum of R-CaMPs is compatible with channel rhodopsins. Green and red GCaMPs have discrete spectra and can be effectively used in dual imaging experiments (Figure 1E). Moreover, GCaMP8 and RCaMP1.07 have very similar kDs (~200 nM), facilitating combinatorial experiments.

**Figure 2:**
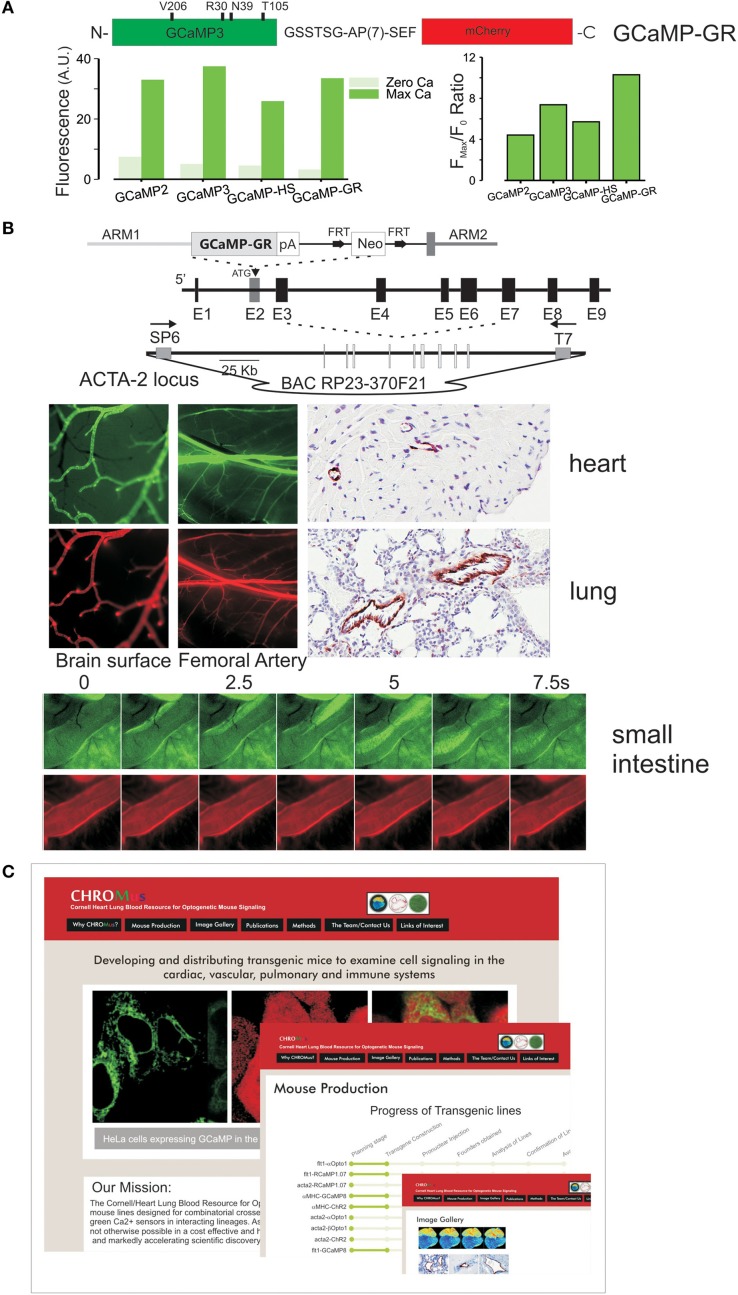
**(A)** Ratiometric GCaMP, **GCaMP-GR**, was created by fusing an improved GCaMP (GCaMP3 with the additional mutations shown) with mCherry, using an optimized linker. Below Ca^2+^ -dependent green fluorescence of GCaMP-GR protein relative to earlier GCaMPs. The F_max_/F_0_ ratio is improved in GCaMP-GR due to a lower F_0_ fluorescence. **(B)** Targeting of GCaMP-GR in mice. Top, a smooth muscle actin bacterial artificial chromosome (ACTA2^BAC^) construct was used to target GCaMP-GR. Middle, smooth muscle–specific green and red fluorescence in vascular smooth muscle, anti-GFP IHC staining. Bottom, green and red fluorescence in smooth muscle of small intestine. **(C)** CHROMus, the Cornell Heart, Lung, Blood Resource for Optogenetic Mouse Signaling, which will create combinatorial mouse strains.

We have recently created a ratiometric GCaMP, GCaMP-GR for green/red, by fusing mCherry to the c-terminus of a high signal GCaMP. The advantages of this approach are that signals can be quantitated and experiments can be targeted by visualizing the bright mCherry expression in cells that have low GCaMP fluorescence at resting Ca^2+^. As shown in Figure 2A, GCaMP-GR, combines the GCaMP3 alanine mutation in calmodulin as well as the M153T, and K203V EGFP mutations that convert GCaMP2 to GCaMP3 (Akerboom et al., 2012), with the four EGFP superfolding mutations that were separately made in GCaMP2 to produce GCaMP-HS (Muto et al., 2011). The resulting GCaMP has a lower resting fluorescence and higher dynamic range than GCaMP3, with a roughly equivalent KD for Ca^2+^ (Figure 2A). We fused mCherry to this sensor to combine bright, Ca^2+^ -independent mCherry fluorescence, enabling easy detection of expressing cells, thereby obviating the problem of minimal GCaMP fluorescence at low Ca^2+^, and enabling ratiometric measurements. The linker after the calmodulin sequence at the c-terminus of GCaMP was systematically varied to maintain full GCaMP dynamic range as well as mCherry brightness. The best result was obtained with a flexible alanine-proline repeat linker (GSSTSG-APAPAPAPAPAPAP-SEF) and GCaMP-GR displays a slightly higher gain that the original modified GCaMP. We used this sensor to create a ratiometric GCaMP-GR mouse, in which GCaMP-GR is placed within an ACTA2 bacterial articifical chromosome and is highly expressed in smooth muscle (Figure 2B). High intensity red fluorescence completely overlaps with green fluorescence, enabling quantitative measurements.

## Genetically encoded optical effectors

Optogenetic effectors, or light–activated effector proteins, represent a parallel, and complementary set of tools, enabling targeted cell activation (Boyden et al., 2005; Miller, 2006; Wang et al., 2007; Fenno et al., 2011; Rein and Deussing, 2012). Similar to genetically encodable Ca^2+^ sensors, these cell actuators can be expressed in a targeted fashion, enabling the activation of discrete biological events, and now comprise a mature and diverse set of tools that enable the activation of a broad array of cellular processes and have advanced to a palette of color-tuned tools. Light–gated channel proteins that depolarize and activate cells have proved to be a major advantage in the study of neural circuits (Boyden et al., 2005; Wang et al., 2007; Witten et al., 2010; Zhang et al., 2010; Lammel et al., 2012; Lee et al., 2012; Narayanan et al., 2012; Warden et al., 2012; Chaudhury et al., 2013; Tye et al., 2013). The applicability of optogenetic effectors has been advanced by the development of fusion products between rhodopsins and G-protein coupled receptors (GPCR); activation of a broad range of “non-excitable” cells is possible through the triggered generation of second messengers (Airan et al., 2009; Gradinaru et al., 2010). Genetic specification of these proteins, combined with the chronic implantation of fiber-optic bundles, enables chronic experiments that reveal complex adaptations within the nervous system (Ung and Arenkiel, 2012; Wang et al., 2012), but also extends the potential utility of these tools to the study of other complex biological processes. The optogenetic effector tool kit now includes channelrhodopsin-2 (ChR2), a 489 nm activated cation channel that depolarizes and activates excitable cells (Zhang et al., 2006), and the rhodopsin/adrenergic receptor fusion proteins opto-α1AR and opto-β 2AR, which generate the intracellular 2nd messengers InsP3/DAG and cAMP, respectively (Airan et al., 2009). As with GCaMPs, optogenetic effectors have been progressively improved, with structurally guided modifications of ChR1 and ChR2 resulting in a lower light activation threshold and increased current outputs (Kleinlogel et al., 2011; Prigge et al., 2012; Lin et al., 2013; Dawydow et al., 2014; Pan et al., 2014).

Moreover, light–activated hyperpolarizing proton pumps mediate cell silencing, extending the range of effective circuit interrogations (Chow et al., 2010). Rhodopsin–based tools are particularly well suited to pairing with red–shifted detectors such as RCaMP1.07, as excitation of the effectors occurs in the range of 480–500 nm and RCaMP1.07 or R-GECO1 are not excited by the effector stimulus. Conversely, the excitation of rhodopsins overlaps with that of the EGFP–based GCaMPs, resulting in an inability to terminate rhodopsin activation while monitoring GCaMP signals. Blue and red–shifted channelrhodopsins have been developed that decrease, but do not eliminate, the activation of effectors by the excitation of GCaMP fluorescence (Prigge et al., 2012). In practice, the rapid activation and desensitization of optogenetic effectors may enable the use of lineage specified rhodopsin proteins with GCaMP–based detectors, despite the spectral overlap, but the pairing of ChR2 and RCaMP avoids this problem.

## A genetic resource for optogenetic effectors and sensors

To date there has been minimal production of mice expressing optogenetic proteins or GECIs, and no targeted effort to produce optically compatible, combinatorial lines. Not suprisingly, most applications have involved viral expression of these proteins, an approach that has several distinct drawbacks including variable and transient expression in target cells, overexpression that could alter normal signaling, and the inability to perform combinatorial experiments. While a few recombinase-based lines have been developed (Witten et al., 2011; Zariwala et al., 2012), these bi-allelic strategies are problematic in that they require complicated crosses, often do not satisfy the stringent requirements of high expression and tight lineage control, and are not an effective combinatorial strategy, since expressing four desired alleles (recombinase plus floxed allele for each optogenetic tool) in a single animal is too inefficient to be practical. Knock-in strategies also have limitations, as the disruption of multiple gene loci may result in poorly predictable alterations in normal physiology. While random insertion of relatively large DNA elements such as BACs may in some circumstances alter local gene expression, it is the technique most likely to yield robust, lineage specific expression in mice that can be simply crossed with other BAC transgenics. Thus, if effector and detector, or multi-wavelength approaches are to be pursued, or these technologies are to be widely deployed in knockout mice, monoallelic lines with strong, tissue specific expression are required. Our experience to date indicates that this approach, combined with careful screening, results in stable lines that can be routinely used as a unique tool to probe complex systems at a molecular scale (Ji et al., 2004; Tallini et al., 2006a,b, 2007, 2009, 2014; Roell et al., 2007; Ledoux et al., 2008; Krasteva et al., 2011; Nausch et al., 2012; Shiba et al., 2012; Sonkusare et al., 2012; Chong et al., 2014; Sonkusare et al., 2014).

The Cornell/National Heart, Lung, Blood Resource for Optogenetic Mouse Signaling (CHROMus) seeks to accelerate the pace at which optogenetic tools (sensors and effectors) are deployed to explore complex biology and disease by developing, publicizing, and distributing key lines that can be exploited by numerous laboratories in different physiological contexts (Figure 2C). The Resource will create and validate lineage specific mouse lines designed for combinatorial use such that the co-expression of optically compatible effectors and sensors, or the expression of sensors with discrete emission wavelengths in interacting lineages (e.g., endothelial and smooth muscle cells), can be achieved with simple, high efficiency crosses. The strategy will enable the combination of spectrally compatible sensor-sensor, or sensor/effector, in specific lineages in a manner unachievable by virally mediated gene transfer. Separate, homozygous lines of lineage specific expressors can be maintained by individual laboratories based on their interest, and combined in simple, high yield breeding strategies, enabling experiments that interrogate complex systems. CHROMus will create and validate 50 mouse lines, communicate their availability, and bank them for distribution to the scientific community, creating a permanent optogenetic resource. All mice will be created on an FVB/NJ—C57BL/2J hybrids and back crossed onto the C57BL/6J strain. The resource will combine several technical breakthroughs made over the past few years including the creation of efficient activators of secondary messengers such as InsP3 and cAMP, brighter and higher dynamic range GECIs, the creation of high-signal low-affinity color-shifted GCaMPs, and the advanced transcriptional control of BAC transgenesis. It will combine these technologies in a targeted, large-scale manner, enabling investigators to more easily realize the promise of optogenetic sensor/effector technology for the investigation of complex biological processes. The resource will develop the first transgenic animals to express the new generation of wave-length shifted, affinity–shifted, and higher gain GECIs, and enable the first systematic combination of optogenetic effectors and high-signal genetic Ca^2+^ detectors. Through the creation, dissemination, and distribution of this state-of-the art resource of signaling mouse lines, CHROMus mice will enable experiments that are not currently feasible. Lines will include those with lineage specific expression of high gain GECIs with discrete emission wavelengths and tuned Ca^2+^ affinities, and optogenetic proteins that enable light–activated cellular depolarization or release of the key second messengers InsP3/diacylglycerol or cAMP. BAC or promoter constructs expressing Ca^2+^ and other biosensors, and light–activated receptors (opto-α1AR, opto-β 2AR) and channels (ChR2) will be created and tested for robust lineage-specific expression. In addition to producing specified lines that have been chosen for their immediate relevance to NHLBI investigators, we will retain the capacity to produce additional lines proposed by NHLBI scientists over the Resource funding period. A Steering Committee with broad NHLBI expertise will approve these additional lines from requests solicited from funded investigators. Thus, the proposed resource will accelerate access to important technologies critical to the understanding and treatment of diseases relevant to the NHLBI.

### Conflict of interest statement

The authors declare that the research was conducted in the absence of any commercial or financial relationships that could be construed as a potential conflict of interest. Supported by NIH R24 HL-120847-01 (Michael I. Kotlikoff).
